# Looking for Hidden Enemies of Metabarcoding: Species Composition, Habitat and Management Can Strongly Influence DNA Extraction while Examining Grassland Communities

**DOI:** 10.3390/biom11020318

**Published:** 2021-02-19

**Authors:** Anna Rucińska, Marcin Olszak, Sebastian Świerszcz, Marcin Nobis, Szymon Zubek, Grzegorz Kusza, Maja Boczkowska, Arkadiusz Nowak

**Affiliations:** 1 Polish Academy of Sciences Botanical Garden, Center for Biological Diversity Conservation in Powsin, Prawdziwka 2, 02-976 Warszawa, Poland; a.rucinska@obpan.pl (A.R.); marcin.olszak@ibb.waw.pl (M.O.); m.boczkowska@ihar.edu.pl (M.B.); anowak@uni.opole.pl (A.N.); 2 Institute of Biochemistry and Biophysics, Polish Academy of Sciences, Pawińskiego 5A, 02-106 Warszawa, Poland; 3 The Franciszek Górski Institute of Plant Physiology, Polish Academy of Sciences, Niezapominajek 21, 30-239 Kraków, Poland; 4 Institute of Botany, Faculty of Biology, Jagiellonian University, Gronostajowa 3, 30-387 Kraków, Poland; m.nobis@uj.edu.pl (M.N.); szymon.zubek@uj.edu.pl (S.Z.); 5 Research Laboratory ‘Herbarium’, National Research Tomsk State University, 634050 Tomsk, Russia; 6 Institute of Biology, University of Opole, Oleska 22, 45-052 Opole, Poland; kuszag@uni.opole.pl; 7 National Centre for Plant Genetic Resources, Plant Breeding and Acclimatization Institute (IHAR)–National Research Institute, Radzików, 05-870 Błonie, Poland

**Keywords:** DNA quality, belowground diversity, graminoid vegetation, Central Europe, roots, DNA extraction

## Abstract

Despite the raising preoccupation, the critical question of how the plant community is composed belowground still remains unresolved, particularly for the conservation priority types of vegetation. The usefulness of metabarcoding analysis of the belowground parts of the plant community is subjected to a considerable bias, that often impedes detection of all species in a sample due to insufficient DNA quality or quantity. In the presented study we have attempted to find environmental factors that determine the amount and quality of DNA extracted from total plant tissue from above- and belowground samples (1000 and 10,000 cm^2^). We analyzed the influence of land use intensity, soil properties, species composition, and season on DNA extraction. The most important factors for DNA quality were vegetation type, soil conductometry (EC), and soil pH for the belowground samples. The species that significantly decreased the DNA quality were *Calamagrostis epigejos*, *Coronilla varia*, and *Holcus lanatus*. For the aboveground part of the vegetation, the season, management intensity, and certain species—with the most prominent being *Centaurea rhenana* and *Cirsium canum*—have the highest influence. Additionally, we found that sample size, soil granulation, MgO, organic C, K_2_O, and total soil N content are important for DNA extraction effectiveness. Both low EC and pH reduce significantly the yield and quality of DNA. Identifying the potential inhibitors of DNA isolation and predicting difficulties of sampling the vegetation plots for metabarcoding analysis will help to optimize the universal, low-cost multi-stage DNA extraction procedure in molecular ecology studies.

## 1. Introduction

### 1.1. Assessing Belowground Biodiversity

The vegetation ecology suffers to some extent because of shortages due to observational restrictions. A critical question of how the community is structured in space and time still remains not fully resolved for the majority of ecosystems as the species data stems mainly from the above ground surveys. A great majority of research, which has focused on niche differentiation, biotic interactions, environmental filtering, species coexistence, functional diversity or typology of plant communities, as well as temporal and spatial changes in vegetation, or the influence of environmental variables on vegetation, considers only the aboveground components of the plant communities as a representative diversity measure e.g., [1,2,3,4,5]. However, in many ecosystems, particularly in stressful habitats, the absolute plant richness and majority of biomass (e.g., 50–90% or even more) is located belowground as roots, bulbs, rhizomes, and shoot bases [6]. This is supposed to be due to persistent belowground meristems, enabling dormancy in the soil without producing aboveground shoots [7,8]. Plants can spread roots farther than shoots [9], via stolons or rhizomes [10], resulting in overlapping root systems and increased species coexistence belowground.

Understanding the community dynamics, spatial and temporal patterns of species distribution and their functional traits or predicting the response of vegetation to environmental changes requires careful examination of belowground plant components [11]. Unfortunately, studies of the belowground part of plant communities are hampered by the problem of a reliable assigning of belowground plant organs to a species. Other restrictions are: (i) seasonality of vegetation cover along with an often limited time window available to sample individuals that can be identified using particular morphological character, e.g., floral characters of plants [12,13]; (ii) the underdevelopment of species in a high competitive community; (iii) destruction of the above part of a species by animals or humans; (iv) problems with identification of cryptic species or hybrids; and (v) the so called ‘bad years’ when the species remains dormant underground and detection problems occur, despite thorough field investigation even for several years. Shoots may be missing in some years but present in others [8,14], resulting in > 30% variation in aboveground richness among years in natural vegetation [15,16,17,18]. Recent development of metabarcoding of environmental DNA (eDNA) and community DNA with the associated cost reduction is gradually making analyses of mixed-species environmental samples more accessible [19,20,21].

### 1.2. Metabarcoding as a Useful Tool for Identifying Belowground Species Richness

DNA metabarcoding as the simultaneous characterization of the whole plant community is suggested as an alternative or complementary method to traditional field research aimed at biodiversity estimation [22,23]. Total DNA extracted from the living belowground plant tissues can considerably improve our understanding of the community organization, dynamics and diversity [6,19]. A growing number of biologists are using metabarcoding DNA for plants detection in a given environmental sample [24,25,26]. The internal transcribed spacer *(ITS2*) subunit region has been often employed for plant metabarcoding as it has potentially high resolution at the genus and species level [26,27]. The application of maturase K (*matK*) and *RuBisCo* (*rbcL*) regions is also considered for that purpose [28]. The majority of studies have been based on analysis of different regions of the chloroplast DNA, *trnL*(*UAA*) intron, encoded *rbcL* gene and nuclear 16S/18S ribosomal RNA genes or *12Smt*DNA to find species composition of different groups of plants in the soil ecosystem [11,19,29,30,31,32].

### 1.3. Factors Affecting the Quality and Quantity of Extracted DNA (Including Environmental and Biochemical)

DNA metabarcoding most commonly refers to DNA extracted from environmental samples [33] which further is used to assay genome regions from one or more species through molecular genetic techniques including PCR, DNA sequencing, and high-throughput sequencing to characterize organisms present in a sample. One of the crucial steps for metabarcoding analysis is the extraction of high-quality DNA in sufficient quantity [34,35,36]. Many plant species produce various secondary metabolites that can interfere with both the extraction of high quality DNA and subsequent PCR analyses [37]. In practice, DNA extraction protocols are very often adjusted to particular plant species or plant tissues to obtain high quality genetic material for downstream analyses [38,39,40,41]. It has proven problematic to recommend a standardized DNA extraction protocol for plants [42]. The extraction of high quality DNA from belowground plant tissues for metabarcoding analysis seems to be more challenging. Assessing the plant richness and true composition in plant communities requires extraction of total DNA from all belowground plant biomasses (roots, bulbs, rhizomes and shoot bases) obtained from soil samples to avoid under-representation or missing any species. However, ‘plant community soil’ contains plant-derived substances, such as humic, fulvic, and tannic acids, and thus contaminate DNA extracted from environmental samples [43,44].

In this study we address the question of what are the most influential factors that determine the quantity and quality of DNA extracted from belowground plant tissues, with a special emphasis placed on the efficiency of indexed PCR dedicated for metabarcoding analysis. We sampled three types of grassland communities (wet, moderately wet, and dry), three types of management intensity within three seasons (spring, summer, and autumn). We applied combined DNA extraction methods, in terms of DNA yield and purity, followed by sets of individually indexed PCRs. Our primary hypotheses were the following: (i) the quality of extracted DNA is hampered mainly by biochemical compounds of some coexisting species; (ii) the most influential species that inhibit the isolation of DNA are rich in carbohydrates and phenolic compounds; (iii) the intensity of management also has a significant impact on the quality and quantity of extracted DNA as it is related to the compactness of soil, increased contamination and accumulation of polysaccharides in belowground organs of plants due to aboveground stress; and (iv) the soil granulometry, particularly the finest particles fraction that effectively hamper the DNA extraction as they cannot be easily removed from the samples.

## 2. Materials and Methods

### 2.1. Study Area and Sampling Design

The surveyed vegetation types are located in central part of Opole region (SW Poland) close to its capital (wet: 50.646886, 17.951331, moderately wet: 50.681413, 17.994983, dry: 50.494326, 18.047414). Each study site was selected in quasi-homogenous stands of recognizable, different vegetation types regarding both site conditions and floristic composition. The vegetation could be classified at alliance level (wet—*Calthion*, moderately wet—*Arrhenatherion*, dry—*Mesobromion*; [45]). The meadows selected for the research differ considerably in their floristic composition. The dominant species in the peaty meadow are: *Cirsium canum*, *C. rivulare*, *Lotus uliginosus*, *Sanguisorba officinalis*, and *Selinum carvifolia*. The brown soil is rich in organic matter, deep and permanently wet with long standing pools and paddles. The moderately wet meadow is composed mainly by *Achillea millefolium*, *Arrhenatherum elatius*, *Plantago lanceolata*, *Ranunculus acris*, *Taraxacum officinale,* and *Trifolium repens*. The brown soil is compacted and moderately fertile with considerable sand fraction. The richest in species is dry xerothermophilous grassland (*Mesobromion*) on ranker, alkaline, shallow, skeletal soil. It is composed mainly of *Bromus erectus*, *Centaurea rhenana*, *Festuca ovina*, *Lotus corniculatus*, *Medicago falcata*, and *Sanguisorba minor*. All the sites were monitored over a long period and are well known in terms of species composition and management.

**Figure 1:**
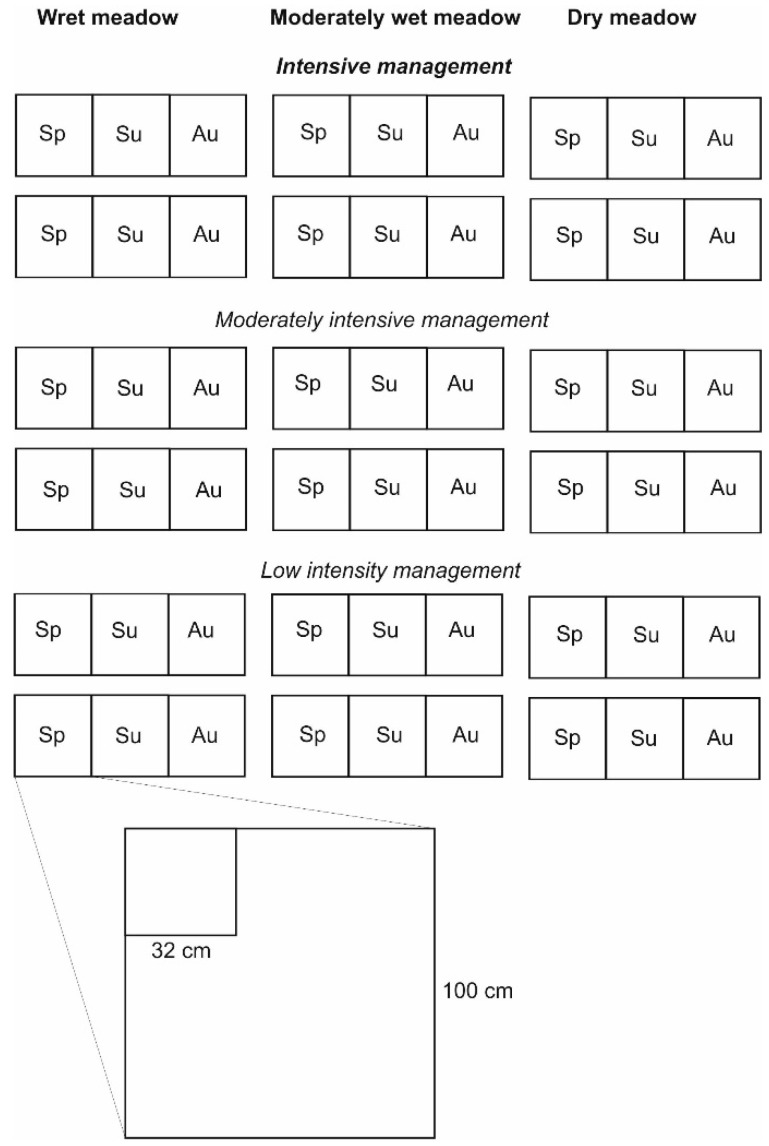
Sampling design. Each plot is divided into two subplots: 1 m^2^ (100 cm × 100 cm) and 0.1 m^2^ (32 cm × 32 cm). Abbreviations: Sp—spring, Su—summer, Au—autumn.

In each site we selected three subareas of different intensity of management (no mowing, mowing twice a year, and intensive mowing (more than 2 per year + trampling). In each subarea we selected six plots in two groups (Figure 1). In order to find the phenological deviation in the aboveground–belowground ratio, two of them were sampled in spring (early April), two in early summer (early June), and two in autumn (September). Each of the six plots were sampled two times in nested subplots to explore the influence of sample size. The size of the two subplots was assigned using the scale differentiation by one order of magnitude as is frequently used in other vegetation ecology studies, particularly in nested-plot design [46,47]. The smaller subplot has the area of 1000 cm^2^ and the larger 10,000 cm^2^ (Figure 1). As a result we obtained 108 samples of different size (54 × 1000 cm^2^, 54 × 10,000 cm^2^), different management intensity and disturbance (36 in low intensity, 36 in medium intensity, and 36 in high intensity), different season (36 for spring, 36 for summer, and 36 for autumn) and different humidity (36 in dry, 36 in moderately wet, and 36 in wet).

### 2.2. Collection of Floristic and Environmental Data

The field studies were conducted in 2018 in grasslands in the Opole Silesia region located in the middle part of Central Europe (temperate climatic zone, *circa* 250 m above sea level, average precipitation *circa* 450 mm/y, average temperature *circa* 9 °C/y; [48]). The plant material was sampled from aboveground and from belowground. For each vegetation plot all the vascular plants were noted. Plant species were recorded according to the percentage cover-abundance scale. Identification and nomenclature of species were conducted according to Rutkowski [49] and Mirek et al. [50].

### 2.3. Extraction, Isolation and Determination of DNA Quality and Quantity

#### 2.3.1. Soil and Plant Samples for DNA Extraction

The top 35 cm of soil layer was collected from two types of plots: 32 cm × 32 cm (1000 cm^2^) and 100 cm × 100 cm (10,000 cm^2^). In total, 108 soil samples were collected from both types of plots which represented belowground plant tissues and, equally, 108 plant samples representing aboveground plant tissue. The soil samples were air-dried (temperature of 25 °C) and sieved (2-mm mesh size) before laboratory analysis. The following physico-chemical and chemical variables of soil were measured according to standard methods: pH in H_2_O and KCl, electrical conductivity (EC), the content of total nitrogen, total and organic carbon, MgO, P_2_O_5_, and K_2_O and bulk density (%). We used a soil texture analysis kit (LaMotte Co., Chestertown, MD, USA) to estimate the proportions of sand, silt, and clay in each soil sample to find out any influence of soil features and contaminants on the quality and quantity of extracted DNA.

#### 2.3.2. DNA Extraction

DNA extraction from belowground and aboveground total biomass of each analyzed plot (Figure 1) consisted of two standard steps: (i) DNA extraction, and (ii) DNA purification. This procedure allowed us to obtain high molecular weight (HMW) DNA from all the analyzed samples suitable for metabarcoding analysis.

(i) DNA extraction

The collected and pre-purified belowground and aboveground plant material samples were crushed in the presence of liquid nitrogen and then mixed. Next, a 0.5 kg sample was taken and subjected to grinding in a blender with the addition of a small amount of liquid nitrogen (LN). The DNA extraction was made from 4 g of belowground and 3 g of aboveground samples of plant tissues by the CTAB-based method [51]. Each sample was mixed with 40 mL of CTAB buffer (20 g CTAB/L, 1.4 M NaCl, 0.1 M Tris–HCl, 20 mM EDTA, 8.5 mM sodium pyrosulphite) and 10 μL proteinase K (20 mg/mL). The samples were incubated for 45 min at 65 °C with shaking. After centrifugation, the supernatants were washed twice with chloroform:isoamyl alcohol (24:1) and treated with RNAse. DNA was precipitated using 1:1 volume of isopropanol, the pellet was washed with ethanol and resuspended in 600 μL of sterile deionized water. Total DNA extracted by CTAB was still heavily contaminated, dark brown in color.

(ii) DNA purification

The DNeasy PowerSoil Kit (Qiagen, Hilden, Germany) was used according to the protocol provided by the manufacturer, including all steps with one modification—short (3 s) vortexing was used instead of bead-beating. The input sample was 200 µL of contaminated DNA obtained in the previously described step. DNA was eluted column with 100 µL sterile deionized water.

#### 2.3.3. Quantity and Quality Measurement of Plant DNA

The quantity and purity of the extracted DNA were measured in a spectrophotometer (NanoDrop One, Thermo Fisher Scientific Inc., Wilmington, DE, USA) based on the formula: Concentration (ng/µL) = (OD260 × 33). The ratios of the absorbance at 260 nm to 280 and 230 nm were used as indicators of contamination of DNA with proteins (A260/A280) and carbohydrates (A260/A230), respectively.

#### 2.3.4. PCR Amplification of DNA

DNA extracted from aboveground and belowground plant tissues was submitted for PCR amplification by using an Eppendorf Thermal Cycler Mastercycler 50a, using indexed primers dedicated for Illumina MiSeq sequencing. Annealing temperature ranging from 52 to 69 °C and denaturation temperature ranging from 93 to 99 °C were optimized for all of primer pairs (Table S1). Amplifications of plant barcoding loci *trnL-trnF* and *rbcL* were completed using the KAPA HiFi Plant DNA polymerase (KAPA Biosystems, Roche, Pleasanton, CA, USA). All reactions were carried out in a 20 μL reaction volume containing 200 ng of template DNA.

### 2.4. Statistical Analysis

The boosted regression trees (BRT) [51] were used to test the relative influence of combination of environment, soil parameters and species occurrences on plant DNA quantity and quality. Full list of predictors are presented in Table 1.

DNA quantity variable was expressed as a continuous variable. The values of DNA quality were transformed into binary values [1—DNA good quality (range of the A260–A230 absorbance ratio for proteins contamination 1.8–2.0 and range of the A260–A280 absorbance ratio for carbohydrates contamination 1.8–2.2), and 0—DNA contamination (ratio outside of the range for DNA good quality)]. We ran three different BRT models for the below- and aboveground samples. In both models we used a set of predictors that included environmental and species occurrence variables. Because the samples were collected from two different sized plots, we included the sample size into each model. Prior to analysis we calculated Spearman’s rank correlation to detect collinearity between the soil parameters. One of each pair of highly correlated variables (r > 0.7) was omitted from further modeling.

The BRT models were fitted using *gbm* [52] and *dismo* [53] in R version 3.5.0 [54]. In order to calibrate the models, we first adjusted the model parameters, which included bag fraction, tree complexity (*tc*), and learning rate (*lr*) [51]. To determine best parametrization, we ran the model for all possible combinations of *lr* = 0.01, 0.005, 0.001, 0.0005; *tr* = 1, 2, 3, 4, 5, and default bag fraction = 0.5. Next, we selected the combination of these parameters with the highest explained deviance based on a minimum of 1000 trees [51]. The models’ parameters can be found in the Supplementary Material (Table S2). Each model was simplified by reducing the number of predictors. Model performance was assessed using explained deviance values, which were between 0 and 100% and a higher value indicates a better performance of the model.

Interpretation of the models was made by assessing relative influence and visualizing the partial dependency plots by the predictors. The relative influence of each variable is scaled so that the sum adds up to 100%. Partial dependence plots were used to visualize the shape of the relationship between DNA quantity or quality and each predictor within the model [55]. Partial dependence functions show the effect of a variable on the response after accounting for the average effects of all other variables in the model [51]. All partial dependence plots for each predictor within the models are shown in the Supplementary Material (Figures S1–S6).

## 3. Results

### 3.1. Model Performance

**Figure 2:**
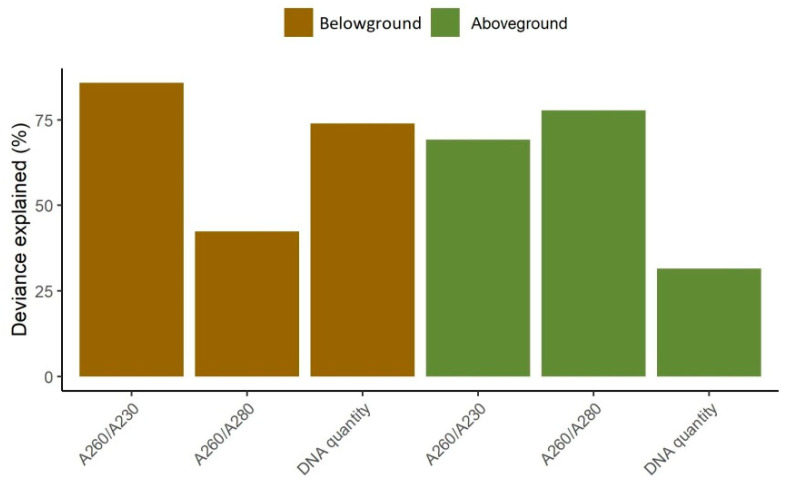
The explained deviance for variables representing DNA quality represented by absorbance ratios A260–A230 and A260–A280 and quantity represented by DNA concentration for belowground (brown bars) and aboveground (green bars) plant material. The values represent models after simplification.

The higher values of deviance explained were observed for DNA quality A260–A230, both for below- and aboveground biomass (85.8 and 69.2%, respectively), quality A260–A280 for aboveground biomass (77.8%) and DNA quantity for belowground biomass (74%), indicating a good model performance (Figure 2). The lowest values of deviance explained were observed for DNA quality A260–A230 for belowground biomass (42.4%) and aboveground DNA quantity (31.5%), that indicate the influence of other variables not included in the model.

### 3.2. Factors Influencing the Extraction of DNA from Belowground Biomass

**Figure 3:**
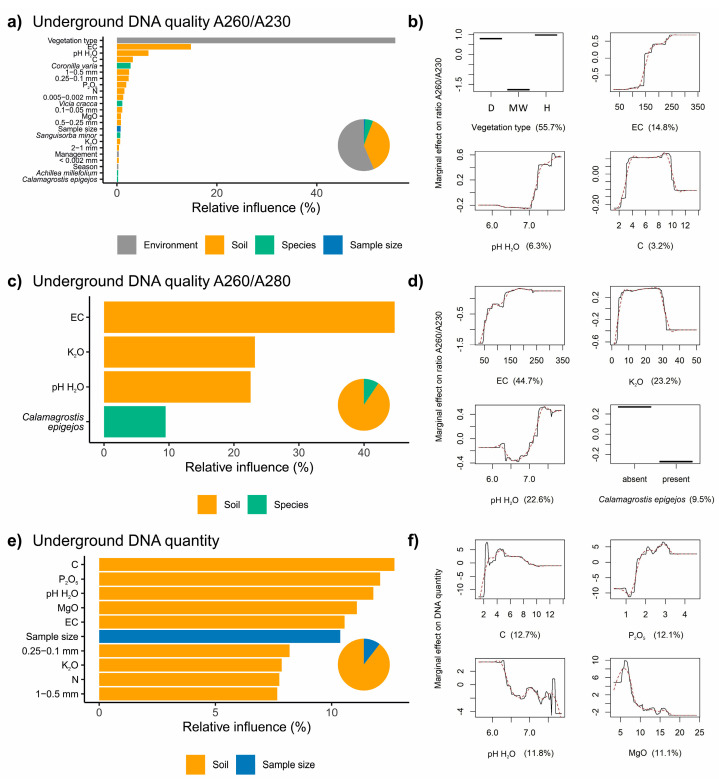
The relative influence (%) and main partial dependence plots showing the marginal relationships between belowground DNA quality: A260–A230 (**a**,**b**), A260–A280 (**c**,**d**), and quantity (**e**,**f**), and predictors after model simplification. Partial dependence plots show the best four predictors. Pie charts represent overall importance of the predictor categories. The full BRT models for DNA quality and quantity and all predictors are shown in Figures S1–S3. Abbreviations: D—dry grassland, MW—moderately wet grassland, W—wet grassland, Sp—spring, Su—summer, Au—autumn; management: L—low intensity, M—medium intensity, H—high intensity. For more details of predictors see Table 1.

For the ratio A260–A230, the relative influence for the predictor categories was, respectively: environment—56.3%, soil—37.9%, species—5%, and sample size—0.8%. The highest relative influence was recorded for vegetation type (55.7%), electrical conductivity (14.8%), and pH (6.3%) (Figure 3a). The model revealed that DNA with the lowest contamination of carbohydrates was in the belowground plant material taken from the dry and wet vegetation types, with soil electrical conductivity >150 µS cm^−1^ and pH > 7 (Figure 3b).

For the ratio A260–A280, the most parsimonious model contains four predictors, with the largest relative influence recorded for soil group predictors, obtaining 90.5% in total, and for *Calamagrostis epigejos* from species group of predictors, obtaining 9.5% (Figure 3c). The model revealed that DNA with the lowest contamination of proteins was in belowground plant material taken from soils with electrical conductivity >150 µS cm^−1^, potassium content between *circa* 5 and 30 mg·kg^−1^, pH > 7 and the absence of *C. epigejos* in the vegetation (Figure 3d). For DNA concentration the largest relative influence was recorded for the group of soil predictors, which reached in total 89.6% (Figure 3e). The partial dependence plots showed that DNA concentration increased with increasing total organic carbon and phosphorus concentration and decreased with increasing pH and magnesium concentration in the soil (Figure 3f).

### 3.3. Factors Influencing the Extraction of DNA from Aboveground Biomass

**Figure 4:**
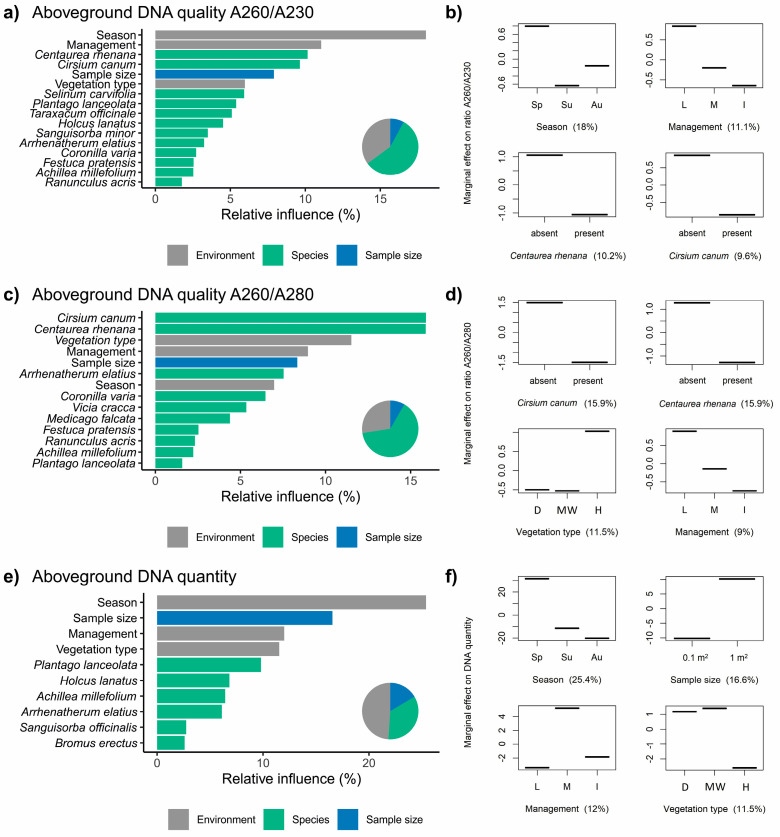
The relative influence (%) and main partial dependence plots showing the marginal relationships between aboveground DNA quality: A260–A230 (**a**,**b**), A260–A280 (**c**,**d**), and quantity (**e**,**f**), and predictors after model simplification. Partial dependence plots show the best four predictors. Pie charts represent overall importance of the predictor categories. The full BRT models for DNA quality and quantity and all predictors are shown in Figures S4–S6. Abbreviations: D—dry grassland, MW—moderately wet grassland, W—wet grassland, Sp—spring, Su—summer, Au—autumn; management: L—low intensity, M—medium intensity, H—high intensity. For more details of predictors see Table 1.

For the ratio A260–A230, the relative influence for the predictor categories was, respectively: environment—35.1%, species—57%, and sample size—7.9% (Figure 4a). The model revealed that DNA with the lowest contamination detected by the 260–230 ratio was in the aboveground plant material taken in the spring, from low managed vegetation and without *Cirsium canum* and *Centaurea rhenana* in the vegetation (Figure 4b).

For the ratio A260–A230, the relative influence for the predictor categories was, respectively: environment—27.5%, species—64.2%, and sample size—8.3% (Figure 4c). The model revealed that DNA with the lowest contamination was in the aboveground plant material with the absence of *C. canum* and *C. rhenana*, taken from wet grasslands with low management intensity (Figure 4d).

For DNA concentration the relative influence for the predictor categories was, respectively: design—48.9%, species—34.5%, and sample size—16.5% (Figure 4e). The model shows that the largest DNA concentration was obtained from material collected in the spring, from a larger sample size, on dry and moderately wet meadows with moderate management intensity (Figure 4f).

### 3.4. DNA Quality and Quantity

**Figure 5:**
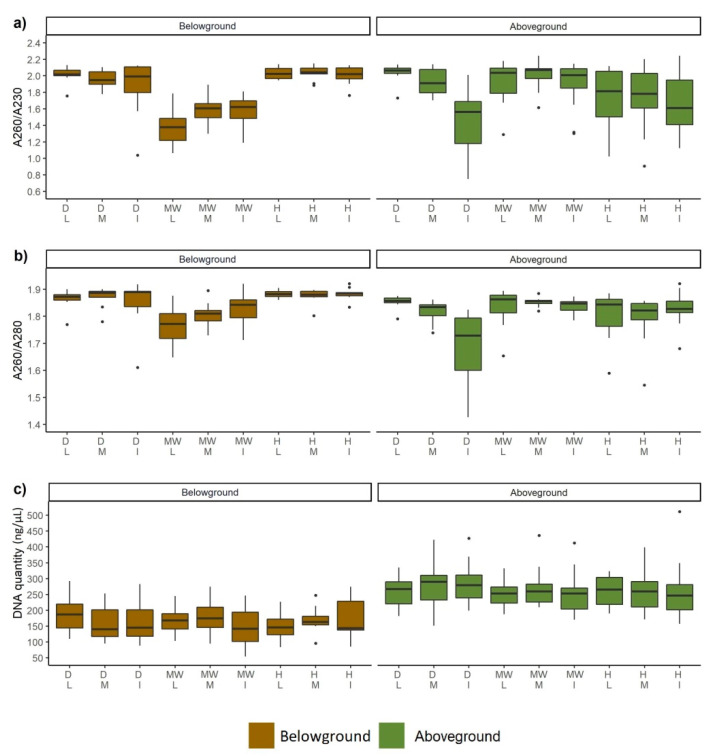
Comparison of quality (**a**,**b**) and yield (**c**) of DNA extracted from various meadow types. The horizontal line denotes the median values, the box indicates the first and third quartiles, and the points indicates outliers. For abbreviations see Figure 3.

**Figure 6:**
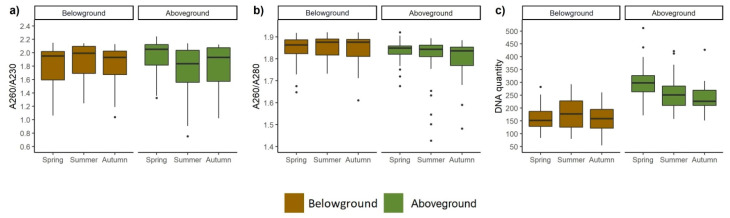
Comparison of quality (**a**,**b**) and yield (**c**) of DNA extracted from various meadows sampled along vegetation season. The horizontal line denotes the median values, the box indicates the first and third quartiles, and the points indicates outliers.

PCR amplification was performed using the DNA after two-step extraction to estimate the effect of DNA extraction efficiency. The obtained results were for *circa* 35% samples of high quality, however for the rest they deviated from the satisfactory levels. The results of DNA quality reflected by the ratios of A260–A280 and A260–A230 for all vegetation types and seasons and the above- and belowground samples are shown in Figure 5 and Figure 6.

The quantity of DNA isolated in different seasons ranged from 98 to 300 ng/µL. The results of the DNA extraction showed that a higher yield of DNA was obtained from samples collected from aboveground tissues in the spring (Figure 6c). The higher amount of DNA was extracted from aboveground (Figure 5c and Figure 6c) than from belowground plant tissue. The best UV absorbance ratio for 260–280 and 260–230 of DNA extracted from belowground tissue was observed for wet meadows. The same ratios indicated the highest level of contamination in moderately wet meadows (Figure 5). The purest DNA from the aboveground tissue was from moderately wet meadows and the most contaminated from wet meadows together with dry, intensively used grassland.

## 4. Discussion

DNA metabarcoding can be applicable in ecosystem-wide studies through assessing species richness and diversity in communities over large spatial scales, though it may be hampered by a number of constraints. In order to address these constraints, we attempted to find all the influential factors in the environment, habitat, and vegetation composition that may significantly affect the quality and quantity of DNA extracted through a two-step isolation protocol.

### 4.1. Plant Chemical Manufacture (Species Composition) Plays a Dominant Role in the Extraction Effectiveness

The plant community composition was the most important factor influencing the quality and quantity of extracted DNA from belowground plant biomass (55.7%) (Figure 3) which was derived from roots, rhizomes, bulbs, and rootstocks. Among species significantly affecting the quantity and quality of DNA, there are species with abundant root systems (e.g., *Coronilla varia*, *Potentilla reptans*, *Polygonum lapathifolium*, *Rumex acetosa*), sometimes tuberous (*Cirsium canum*), with thick taproots (e.g., *Centaurea rhenana*, *Plantago lanceolata*, *Selinum carvifolia*, *Taraxacum officinale*), thick rhizomes and stolons, with densely branching fibrous root system (*Anthoxanthum odoratum*, *Festuca pratensis*, *Holcus lanatus*, *Calamagrostis epigejos*) or robust rootstocks (e.g., *Sanguisorba officinalis*, *S. minor*). Such organs have a large contribution in the belowground biomass, sometimes potentially greater than their share in the aboveground biomass. Hence, as the belowground organs are often storage tissues for resources, they are a large depot for carbohydrates and other secondary metabolites that can strongly affect the DNA extraction [56].

We found that the highest amount of contaminants detected by the A260–A230 ratio concerned the moderately wet vegetation type of grassland. This acidic habitat is also poor in nitrogen, phosphorus, and total organic carbon. Other studies found high tannin concentrations in plants occurring in habitats with low soil fertility and low pH [57]. Thus, our observed pattern of quality DNA could also be driven by a high level of tannin in plant belowground tissue. Among the species contributing to the moderately wet community are *Holcus lanatus*, *Rumex acetosa*, *Festuca rubra*, *Taraxacum officinale*, *Anthoxanthum odoratum*, *Cirsium arvense*, *Arrhenatherum elatius*, and *Calamagrostis epigejos*. Their roots, bulb, and rootstock are known to contain many secondary compounds [58,59,60], thus they can potentially interfere in the DNA extraction.

Due to the fact that the community composition has a major impact on the soil habitat and the rest of the biodiversity that resides within the belowground matrix [61], the high influence of vegetation type on the quality of DNA extracted from belowground biomass is potentially involved with roots exudates and microorganisms in the vicinity of the root [62]. Some species exhibit an increased amount of exudates in a specific environment. For example, low pH of soil can increase twice the amount of malate secreted by the roots of *Holcus lanatus* [63], theoretically suggesting some contribution in the lower quality of DNA from moderate wet meadow.

One of the most abundant species of moderately wet grassland was *C. epigejos*, whose presence in the plot is positively correlated with a high level of DNA contamination (Figure 3c). It was found that the powerful competitive ability of this species is related to the effective storage of nitrogen compounds in the roots [64]. A high content of fructans (20–25% of dry biomass) and starch (up to 14% of dry biomass) in rhizomes [65] suggested that these compounds could be one of the main source of contamination in DNA extract from moderately wet meadows, especially when bearing in mind the fairly high contribution of this species in many plots. 

DNA obtained from plots with *Coronilla varia* also exhibited lower quality (Figure 3a). *C. varia* is a plant rich in essential oils with confirmed cytotoxic properties [66]. Such plants—rich in essential oils—can affect the quality and quantity of DNA isolation [67,68]. *C. varia* is also a plant rich in tannins. The condensed tannin concentration in *C. varia* was 16.0 (g/kg of dry weight) and it was isolated from active fractions of the crude roots extracts [69]. Interestingly, *C. varia* was observed only on dry meadows, suggesting the relatively strong effect of its root compounds on DNA extraction from bulked plant tissue.

### 4.2. Soil Properties also Play an Important Role in the Extraction Effectiveness

The lower quality and quantity of DNA was also influenced by the sample surface (Figure 3e). Belowground material collected from either the 1000 or 10,000 cm^2^ area was processed in a way that minimized the loss of species information and to avoid the underrepresentation of fine roots which make up around 90% of the root system total length [70]. Elimination of all soil particles was impossible due to the soil structure and high rooting density. Hence a relatively low yield of DNA from the 10,000 cm^2^ area can be attributed to higher contamination by soil particles which may have impeded the DNA extraction process. These mainly include clay parts and floated soils, sand, decaying organic matter (plant but also soil fauna), including humic substances (HS) [71].

Soil particles, together with species composition, determine the soil properties which were also very important factors for DNA purity and quantity from belowground biomass in our study (Figure 3a–f). Increases of EC > 150 μS·cm^−1^ and pH > 7 improve the DNA quality. The highest amount of contamination was observed for moderately wet meadows. It is worth noting the multi-compound properties of lysis solution during extraction of DNA from belowground bulked plant tissue, related to both plant and soil origin. Among the soil particles HS are often described as contaminants of DNA extraction [72]. In the case of plants, the direct effects of HS on their physiology are a consequence of the flow of HS into the apoplast, the changes in proteins transport and hormone-like effects [73], therefore contamination of plant tissue with HS is unavoidable. Physicochemical analogy with the nucleic acids make them coextracted with DNA [74]. Furthermore, humic molecules can be larger than the molecular weight cutoff of centrifugal filters used in DNA purification, causing them to remain in the aqueous phase. DNA molecules could also form complexes with HS, therefore both are eluted together.

The high amount of HS in moderately wet meadow is related to the low pH of this habitat as stable HS occur in disrupted form in an alkaline environment, and only in acidic habitats do HS aggregate and create strong bonding [75]. Dry and wet meadows are richer in ions (EC > 150 μS·cm^−1^), which can be a potential target for bonding properties of HS. We observed in our study the positive correlation of the effectiveness of DNA extraction with the higher level of phosphate and electrolytic conductivity, of which the latter is related to the salt concentration in the soil [76]. However, the higher amount of potassium and magnesium cations negatively correlated with the yield of DNA (Figure 3e,f). Other research has shown that pH of the soil and the lysis buffer are potentially the most influential factors in DNA extraction from soil [77]. The results of our study confirmed that the level of pH < 7 correlates with a higher level of contamination and lower yield of DNA (Figure 3a–c).

The contamination in moderately wet meadows could also come from the adsorption of DNA on clay minerals (CM). Saeki et al. [78] have suggested that DNA adsorption on CM occurs via two mechanisms: direct bonding of the phosphate group at the end of the DNA molecule to the hydroxyl (OH) groups of CM, and the association of DNA molecules with the surface of negatively charged CM via a bridging of inorganic cations. It is also confirmed by Shen et al. [79] who proved that the presence of divalent cations (Ca^2+^) in solutions greatly enhanced (even irreversibly) the adsorption of nucleic acids on CM-related particles when compared to monovalent cations (Na^+^). As Saeki et al. [78] suggest, this first phenomenon is related to the assumption that inorganic anions (phosphate, selenite, and arsenate) sorb on CM via ligand-exchange reactions possibly occurring between the phosphate groups of the DNA molecule and the OH groups of the CM particles. In the second mechanism, DNA molecules associate with the surface of negatively charged CM via a bridging of inorganic cations, even around pH 8. Additionally, this study indicated that DNA adsorption on CM decreased with increasing suspension pH solution, which is fully in line with our results the acidic habitat of moderately wet meadows caused the lower quality of DNA.

All the above-mentioned facts explain the high level of contamination in moderately wet meadows. The relatively low contamination effect of Ca^2+^, Mg^2+^, and phosphate was covered by the acidic character of the soil and thus the adsorption activity of HS and CM to DNA. The contamination effect of a high amount of Ca^2+^ and Mg^2+^ in dry meadows was partly reduced by alkaline habitat (mean 7.47) and a higher level of EC and phosphate resulting in relatively better purification of DNA.

### 4.3. Aboveground, the Species Composition Is the Most Influential

The results of the analyses showed that the presence in plots of species such as *Centaurea rhenana*, *Cirsium canum*, and others such as *Selinum carvifolia*, *Taraxacum officinale* and *Holcus lanatus*, was correlated with the occurrence of difficulties in DNA isolation. This may suggest a link to the chemical composition of these plants and the presence of metabolites that may undergo unpredictable biochemical transformations and strongly contaminate the DNA.

*Cirsium canum* and *Centaurea rhenana* are known to be rich in phenolic compounds [80]. *C. canum* contains flavonoids and phenolic acids, taraxasterol acetate, aliphatic hydrocarbons [81,82], which is why the species is known to reveal strong antibacterial properties on gram-positive strains [82]. *C. rhenana* (=*C. stoebe*; =*C. maculosa*) is also known to have strong phytotoxic and antibacterial properties; it contains terpenoids and catechins [83,84,85].

An unusual response was revealed for *Vicia cracca*. This species inhibits the quality of the DNA only for the aboveground parts of the plant community; the belowground samples were related to the best quality DNA. The species is in fact mainly present in the aboveground biomass as the root system is not abundant, but the leaves and branching stems are quite plenteous. Regarding the plant morphology and organ architecture, a similar situation is found for *Holcus lanatus* that also has a detrimental effect on DNA only for the aboveground samples. This plant is known to contain a great abundance of polysaccharides, aconite acid, and aldehydes [86].

Without properly designed and conducted biochemical analyses it is difficult to indicate the most influential specific compounds. We can only—based on the biochemical profile of these species taken from literature—assume that they are able to infer the extraction procedure because of the relatively high amount of polyphenols, polysaccharides, terpenes, and their derivatives.

### 4.4. Season and Stress Play an Important Role in Extraction of High Quality DNA from the Aboveground Biomass

The two critical factors affecting the DNA isolation are the age of the plant tissue and its type (leaf, stem, etc.) [87]. It is commonly known that young and fresh plant tissues can provide DNA of the best quality and quantity due to the higher amount of young cells with less deposition of starches and secondary metabolites [88,89,90]. DNA from mature leaves, woody stems, etc., is of low quality and the concentrations obtained are low due to the presence of high concentrations of polyphenols, tannins, polysaccharides and other secondary metabolites that can attach strongly to DNA [87].

Seasonal patterns of carbohydrate and nutrient concentrations in the stems and leaves are well documented across many plant species. In many cases, the resorption and translocation of plant secondary metabolites can be important strategies for perennial plants to maximize resource use across multiple growing seasons [91,92]. Seasonal changes in secondary metabolite content also play a crucial role in plant defense against herbivores through toxic and feeding deterrent effects, as well as the attraction of natural enemies of herbivores [93,94,95]. An example is *Taraxacum officinale* that significantly increases secondary metabolite content and reveals strong seasonal variation in root latex chemistry [93].

The seasonality of the plant community has an apparent reflection in the condition of the plant tissue due to biological processes that are undergone during the life cycle and also because of the seasonal variations in habitat conditions. All these constraints make the extraction efficiency at the accepted quality from the autumn, but also some of the summer samples, scarcely achievable (Figure 4).

There is the commonly accepted view that the intensity of stress and disturbance influence the amount of secondary metabolites in plants, e.g., [96,97]. Plants have developed a variety of strategies for defending against herbivores. Among the most influential secondary metabolites, hydroxamic acids [98], tannins [99], cyanogenic glycosides [100] and alkaloids are reported, particularly in grasses that are the most important component of the meadows in our study area [96]. Another example of a reactive substance that might be involved in hampering the extraction procedure is jasmonic acid and hydrogen cyanide that are synthesized and released by plants after injury [101,102,103]. Additionally, terpenes and tannins and terpenoids are synthesized by the plant as a part of the defense system against herbivores [104,105]. The belowground part of the plant also induces the intensive production of secondary metabolites after wounding [106]. The whole plant synthesizes responsive secondary metabolites which can hinder the DNA extraction in the samples from intensively managed grasslands where the cutting and trampling was most severe.

## 5. Conclusions

Molecular ecology methods have been recognized as one of the most promising approaches in the exploration of community structure, particularly its belowground part. As we apparently evidenced, a number of environmental, ecological, and anthropogenic factors significantly influence the quality and quantity of the extracted DNA. Thus, the final results of particular study on species composition, regardless whether the below-, above-part, or the whole vegetation, can be strongly affected. Depending on the abundancy of a particular species and its chemical composition, even one species can effectively hamper the isolation of DNA. The more diverse the vegetation, the higher the risk that the sample includes the biomass impeding the procedure. Among the species that strongly correlate with highly contaminated DNA were fairly common species in Central European grasslands such as *Centaurea rhenana*, *Cirsium canum*, *Taraxacum officinale*, and *Holcus lanatus*. Additionally, the anthropogenic impact can negatively influence the acquisition of proper DNA. The plants strongly respond to intensive management and produce an array of secondary metabolites. Moreover, they can change their allocation due to frequent cutting, thus effectively hampering the extraction of DNA both in above- or belowground samples. Additionally, the sample size was of particular importance as the large ones were barely possible to be adequately cleaned.

Certainly we do not want to dissuade from applying the metabarcoding analyses when examining the real composition of the plant community. However, we believe that it is advisable to identify any potential risk factors that can spoil the results of well-designed research. We have attempted to highlight all the physico-chemical constraints and to explain the possible biochemical interactions in order to improve planning and help in avoiding the dispensable risks. Keeping in mind all the possible impediments related to the research design, species composition, environmental factors and human activity on the study area, it is workable to plan a 2-, 3- or even more step procedure of DNA extraction and to applicate additional, indispensable amendments and refinements of the isolation protocol.

## Figures and Tables

**Table 1:** List of predictors used in this study.

Predictor Category	Predictor	Type	Description
Environment	Vegetation type	categorical	dry grassland
			moderately wet grassland
			wet grassland
	Season	categorical	spring
			summer
			autumn
	Management	categorical	low intensity
			medium intensity
			high intensity
Area	Sample size	categorical	0.01 m^2^
			1 m^2^
Soil parameters	pH H_2_O	continuous	pH measured in H_2_O
	pH KCl	continuous	pH measured in KCl
	EC	continuous	electrical conductivity (μS·cm^−1^)
	CaCO_3_	continuous	calcium (mg·kg^−1^ soil dry)
	C	continuous	total organic carbon (%)
	N	continuous	total nitrogen (% soil dry)
	MgO	continuous	magnesium (mg·kg^−1^ soil dry)
	P_2_O_5_	continuous	phosphorus (mg·kg^−1^ soil dry)
	K_2_O	continuous	potassium (mg·kg^−1^ soil dry)
	2–1 mm	continuous	soil fraction 2–1 mm (%)
	1–0.5 mm	continuous	soil fraction 1–0.5 mm (%)
	0.5–0.25 mm	continuous	soil fraction 0.5–0.25 mm (%)
	0.25–0.1 mm	continuous	soil fraction 0.25–0.1 mm (%)
	0.1–0.05 mm	continuous	soil fraction 0.1–0.05 mm (%)
	0.05–0.02 mm	continuous	soil fraction 0.05–0.02 mm (%)
	0.02–0.005 mm	continuous	soil fraction 0.02–0.005 mm (%)
	0.005–0.002 mm	continuous	soil fraction 0.005–0.002 mm (%)
	< 0.002 mm	continuous	soil fraction < 0.002 mm (%)
Species	Species occurrence	binary	species occurrence presence/absence (1/0)

## Data Availability

The data presented in this study are available on request from the corresponding author.

## Supplementary Materials

The following are available online at https://www.mdpi.com/2218-273X/11/2/318/s1, Table S1: Indexed primer sequences for each locus. Core sequences primers for *rbcL* are from Hollingsworth et al. [107] and *trnL-trnF* are from the study by Taberlet et al. [108]; Table S2: Model parameters for each DNA quality (measured as ratio A260–A230 and A260–A280) and DNA quantity (measured as DNA concentration) aboveground and belowground samples. During model calibration we tested different combinations of *lr* = 0.01, 0.005, 0.001, 0.0005; *tr* = 1, 2, 3, 4, 5 and default bag fraction = 0.5. For max trees we fixed its value in 10,000, except in aboveground DNA concentration, where we fixed to 20,000. Model optimal parameters setting were selected based on a minimum of 1000 trees obtained [51] and the highest explained deviance; Figure S1: Partial dependence plots showing the marginal relationships of belowground DNA quality shown as ratio A260–A230 for all predictors of simplified BRT model; Figure S2: Partial dependence plots showing the marginal relationships of belowground DNA quality shown as ratio A260–A280 for all predictors of simplified BRT model; Figure S3: Partial dependence plots showing the marginal relationships of belowground DNA concentration for all predictors of simplified BRT model; Figure S4: Partial dependence plots showing the marginal relationships of aboveground DNA quality shown as ratio A260–A230 for all predictors of simplified BRT model; Figure S5: Partial dependence plots showing the marginal relationships of aboveground DNA quality shown as ratio A260–A280 for all predictors of simplified BRT model; Figure S6: Partial dependence plots showing the marginal relationships of aboveground DNA concentration for all predictors of simplified BRT model.
