# Supplementary material for: Looking for Hidden Enemies of Metabarcoding: Species Composition, Habitat and Management Can Strongly Influence DNA Extraction while Examining Grassland Communities
Source: Biomolecules. 2021 Feb 19;11(2):318. doi: 10.3390/biom11020318 (PMC7921978; doi:10.3390/biom11020318)
Supplement: Supplementary file 1 [file biomolecules-11-00318-s001.pdf]

Table S1. Indexed primer sequences for each locus. Core sequences primers for *rbcL* are from Hollingsworth et al.[107] and *trnL-trnF* are from the study by Taberlet et al. [108].

region	Primer name	Sequence
	trnL-FF_bc1_F	TCGTCGGCAGCGTCAGATGTGTATAAGAGACAGTAACTCTGGGTTCAAGTCCCTCTATCCC
	trnL-FF_bc2_F	TCGTCGGCAGCGTCAGATGTGTATAAGAGACAGTGAGTCAGGGTTCAAGTCCCTCTATCCC
	trnL-FF_bc3_F	TCGTCGGCAGCGTCAGATGTGTATAAGAGACAGTAGCATCGGGTTCAAGTCCCTCTATCCC
	trnL-FF_bc4_F	TCGTCGGCAGCGTCAGATGTGTATAAGAGACAGAGTCGAGAGGTTCAAGTCCCTCTATCCC
	trnL-FF_bc5_F	TCGTCGGCAGCGTCAGATGTGTATAAGAGACAGCTACAGACGGTTCAAGTCCCTCTATCCC
	trnL-FF_bc6_F	TCGTCGGCAGCGTCAGATGTGTATAAGAGACAGAGCTAGTCGGTTCAAGTCCCTCTATCCC
	trnL-FF_bc7_F	TCGTCGGCAGCGTCAGATGTGTATAAGAGACAGATCACGACGGTTCAAGTCCCTCTATCCC
trnLF	trnL-FF_bc8_F	TCGTCGGCAGCGTCAGATGTGTATAAGAGACAGAGCGTATGGGTTCAAGTCCCTCTATCCC
	trnL-FRTab_bc1_R	GTCTCGTGGGCTCGGAGATGTGTATAAGAGACAGCCATAGGAATTTGAACTGGTGACACGAG
	trnL-FRTab_bc2_R	GTCTCGTGGGCTCGGAGATGTGTATAAGAGACAGAGGACATTATTTGAACTGGTGACACGAG
	trnL-FRTab_bc3_R	GTCTCGTGGGCTCGGAGATGTGTATAAGAGACAGAGACTGACATTTGAACTGGTGACACGAG
	trnL-FRTab_bc4_R	GTCTCGTGGGCTCGGAGATGTGTATAAGAGACAGGTACTGCAATTTGAACTGGTGACACGAG
	trnL-FRTab_bc5_R	GTCTCGTGGGCTCGGAGATGTGTATAAGAGACAGAATGTTCTATTTGAACTGGTGACACGAG
	trnL-FRTab_bc6_R	GTCTCGTGGGCTCGGAGATGTGTATAAGAGACAGTAGCGCTAATTTGAACTGGTGACACGAG
	trnL-FRTab_bc7_R	GTCTCGTGGGCTCGGAGATGTGTATAAGAGACAGGCTATCTCATTTGAACTGGTGACACGAG
	RbcL-aafF_bc1_F	TCGTCGGCAGCGTCAGATGTGTATAAGAGACAGAATATGGGATGTCACCACAAACAGAGACTAAAGC
	RbcL-aafF_bc2_F	TCGTCGGCAGCGTCAGATGTGTATAAGAGACAGACATGCATATGTCACCACAAACAGAGACTAAAGC
	RbcL-aafF_bc3_F	TCGTCGGCAGCGTCAGATGTGTATAAGAGACAGCGCGAAATATGTCACCACAAACAGAGACTAAAGC
	RbcL-aafF_bc4_F	TCGTCGGCAGCGTCAGATGTGTATAAGAGACAGGATGAGGGATGTCACCACAAACAGAGACTAAAGC
	RbcL-aafF_bc5_F	TCGTCGGCAGCGTCAGATGTGTATAAGAGACAGACCATAGGATGTCACCACAAACAGAGACTAAAGC
	RbcL-aafF_bc6_F	TCGTCGGCAGCGTCAGATGTGTATAAGAGACAGCTCCGCAAATGTCACCACAAACAGAGACTAAAGC
	RbcL-aafF_bc7_F	TCGTCGGCAGCGTCAGATGTGTATAAGAGACAGTCCAGGTAATGTCACCACAAACAGAGACTAAAGC
rbcL	RbcL-aafF_bc8_F	TCGTCGGCAGCGTCAGATGTGTATAAGAGACAGCGGATTCAATGTCACCACAAACAGAGACTAAAGC
	rbcLajf634R_bc1_R	GTCTCGTGGGCTCGGAGATGTGTATAAGAGACAGCTAAACAGGAAACGGTCTCTCCAACGCAT
	rbcLajf634R_bc2_R	GTCTCGTGGGCTCGGAGATGTGTATAAGAGACAGACTGGTGTGAAACGGTCTCTCCAACGCAT
	rbcLajf634R_bc3_R	GTCTCGTGGGCTCGGAGATGTGTATAAGAGACAGACACCTAGGAAACGGTCTCTCCAACGCAT
	rbcLajf634R_bc4_R	GTCTCGTGGGCTCGGAGATGTGTATAAGAGACAGACAGTGTAGAAACGGTCTCTCCAACGCAT
	rbcLajf634R_bc5_R	GTCTCGTGGGCTCGGAGATGTGTATAAGAGACAGACGAAAGAGAAACGGTCTCTCCAACGCAT
	rbcLajf634R_bc6_R	GTCTCGTGGGCTCGGAGATGTGTATAAGAGACAGCCACAGGTGAAACGGTCTCTCCAACGCAT
	rbcLajf634R_bc7_R	GTCTCGTGGGCTCGGAGATGTGTATAAGAGACAGCCGATCTTGAAACGGTCTCTCCAACGCAT

Table S2. Model parameters for each DNA quality (measured as ratio A260/A230 and A260/A280) and DNA quantity (measured as DNA concentration) aboveground and belowground samples. During model calibration we tested different combinations of *lr* = 0.01, 0.005, 0.001, 0.0005; *tr* = 1, 2, 3, 4, 5 and default bag fraction = 0.5. For max trees we fixed its value in 10,000, except in aboveground DNA concentration, where we fixed to 20,000. Model optimal parameters setting were selected based on a minimum of 1,000 trees obtained [52] and the highest explained deviance.

**Sample part**	**Explained variable**	***lr***	***tc***	**max trees**	**number of trees**
Aboveground	A260/A230	0.005	5	10,000	1550
	A260/A280	0.01	3	10,000	1150
	DNA concentration	0.0005	5	20,000	11250
Belowground	A260/A230	0.001	5	10,000	3350
	A260/A280	0.0005	5	10,000	3250
	DNA concentration	0.01	5	10,000	1550

Abbreviations for figures S1–S6

Vegetation type: D – dry grassland, MH – moderately wet grassland, H – humid wet; **season**: Sp – spring, Su – summer, Au – autumn; **management**: L – low intensity, M – medium intensity, I – high intensity. For more details see Table 1.

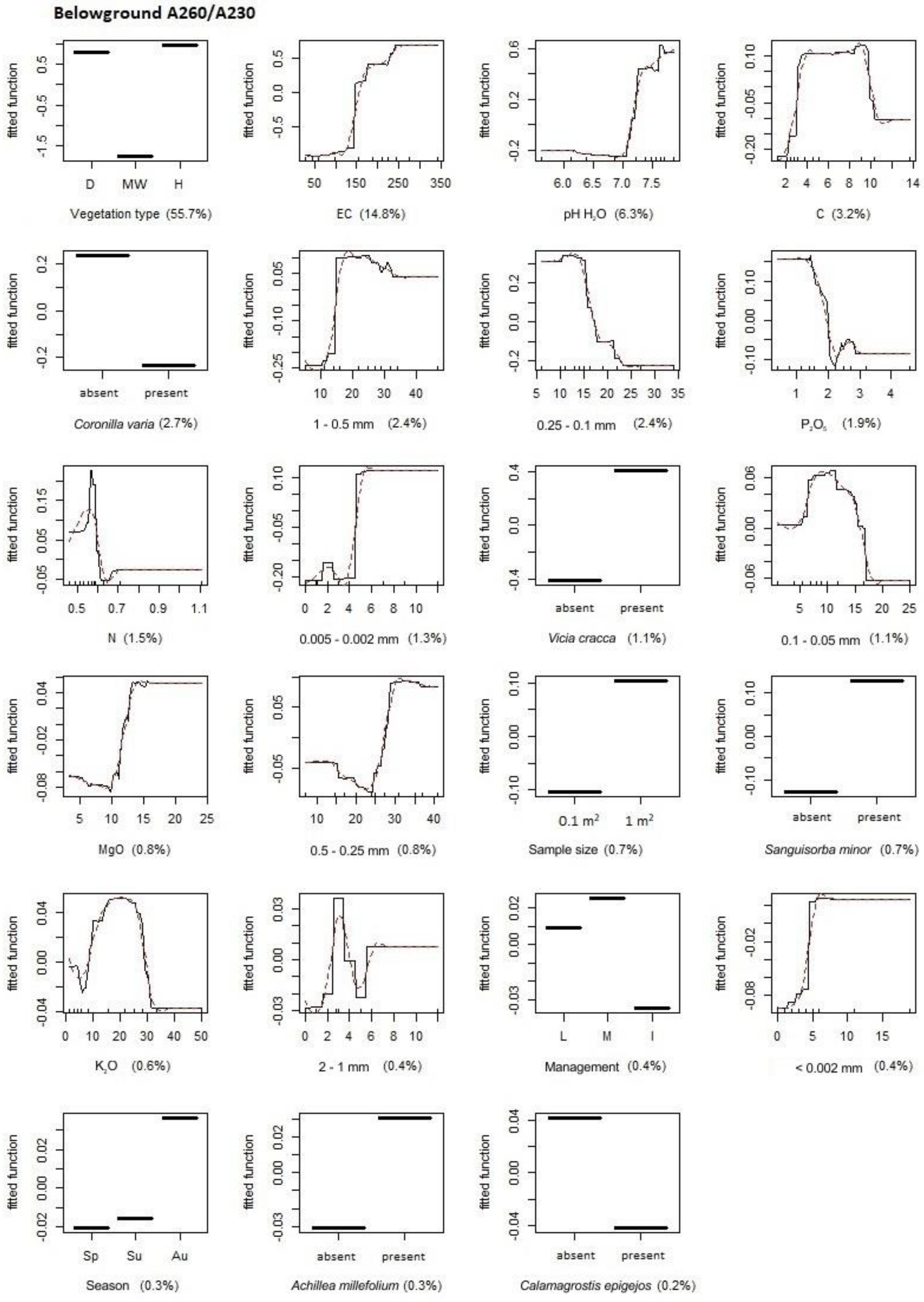

Fig. S1. Partial dependence plots showing the marginal relationships of belowground DNA quality shown as ratio A260/A230 for all predictors of simplified BRT model.

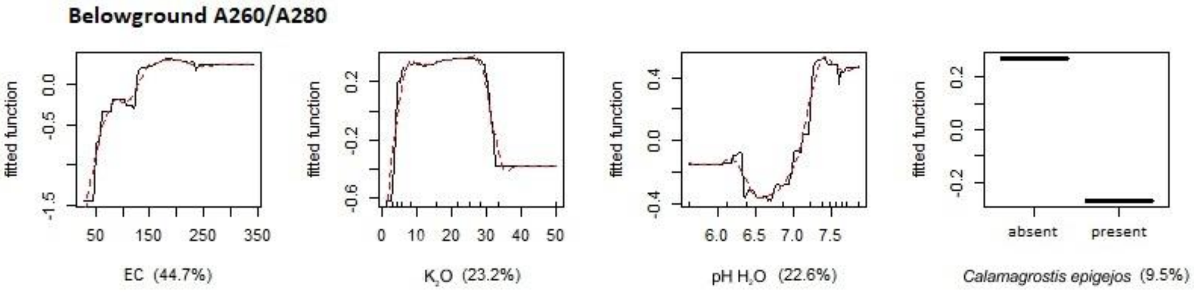

Fig. S2. Partial dependence plots showing the marginal relationships of belowground DNA quality shown as ratio A260/A280 for all predictors of simplified BRT model.

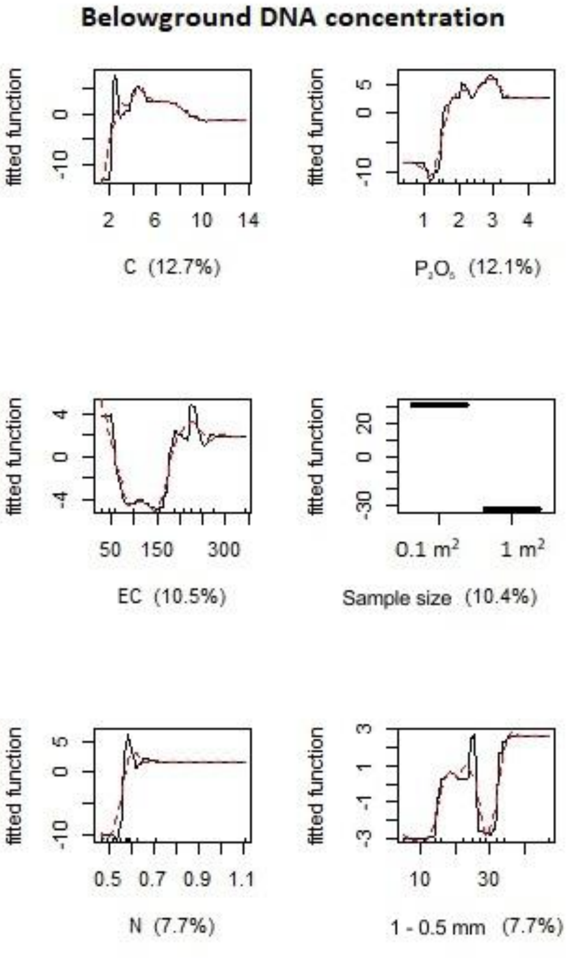

Fig. S3. Partial dependence plots showing the marginal relationships of belowground DNA concentration for all predictors of simplified BRT model.

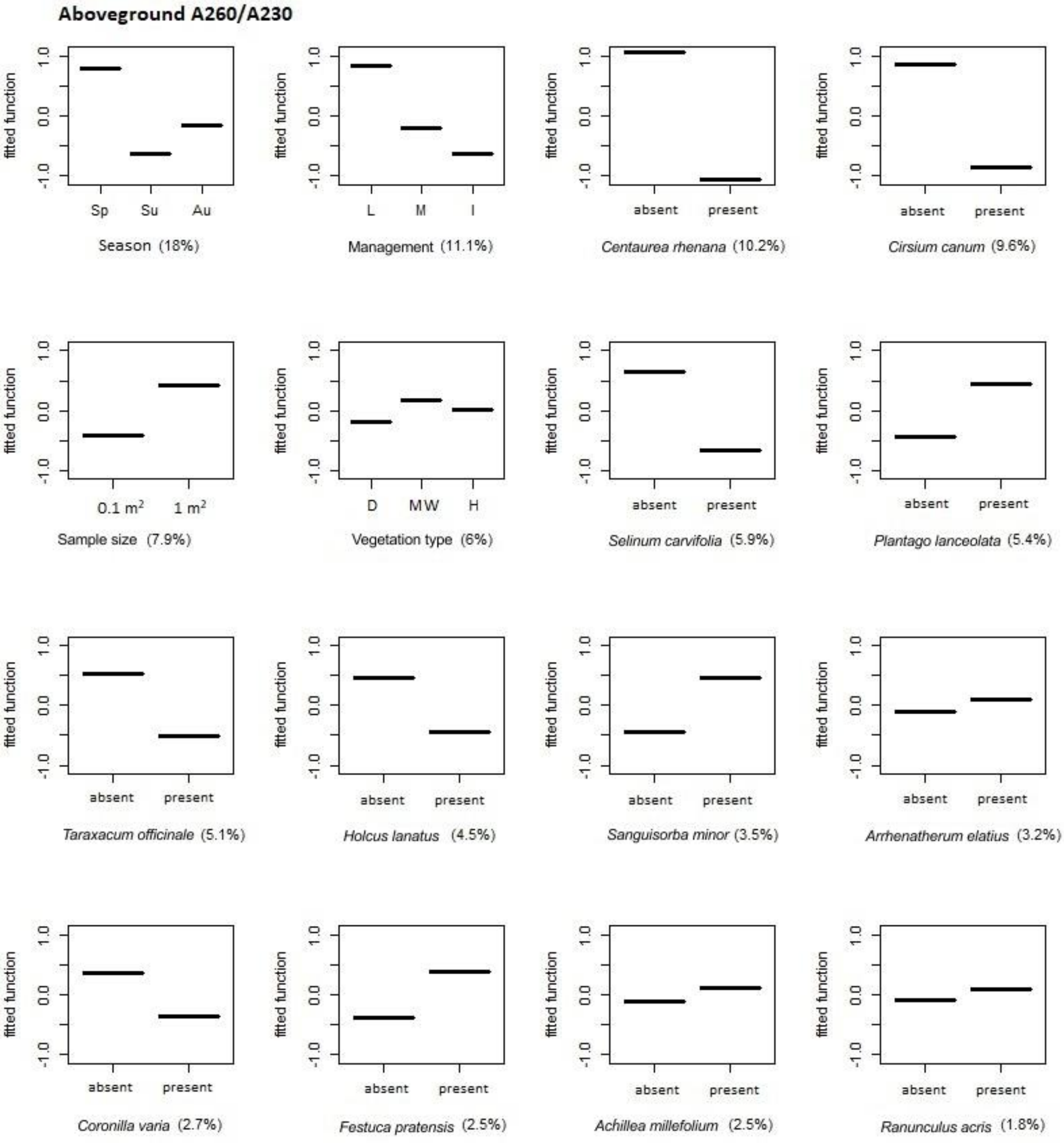

Fig. S4. Partial dependence plots showing the marginal relationships of aboveground DNA quality shown as ratio A260/A230 for all predictors of simplified BRT model.

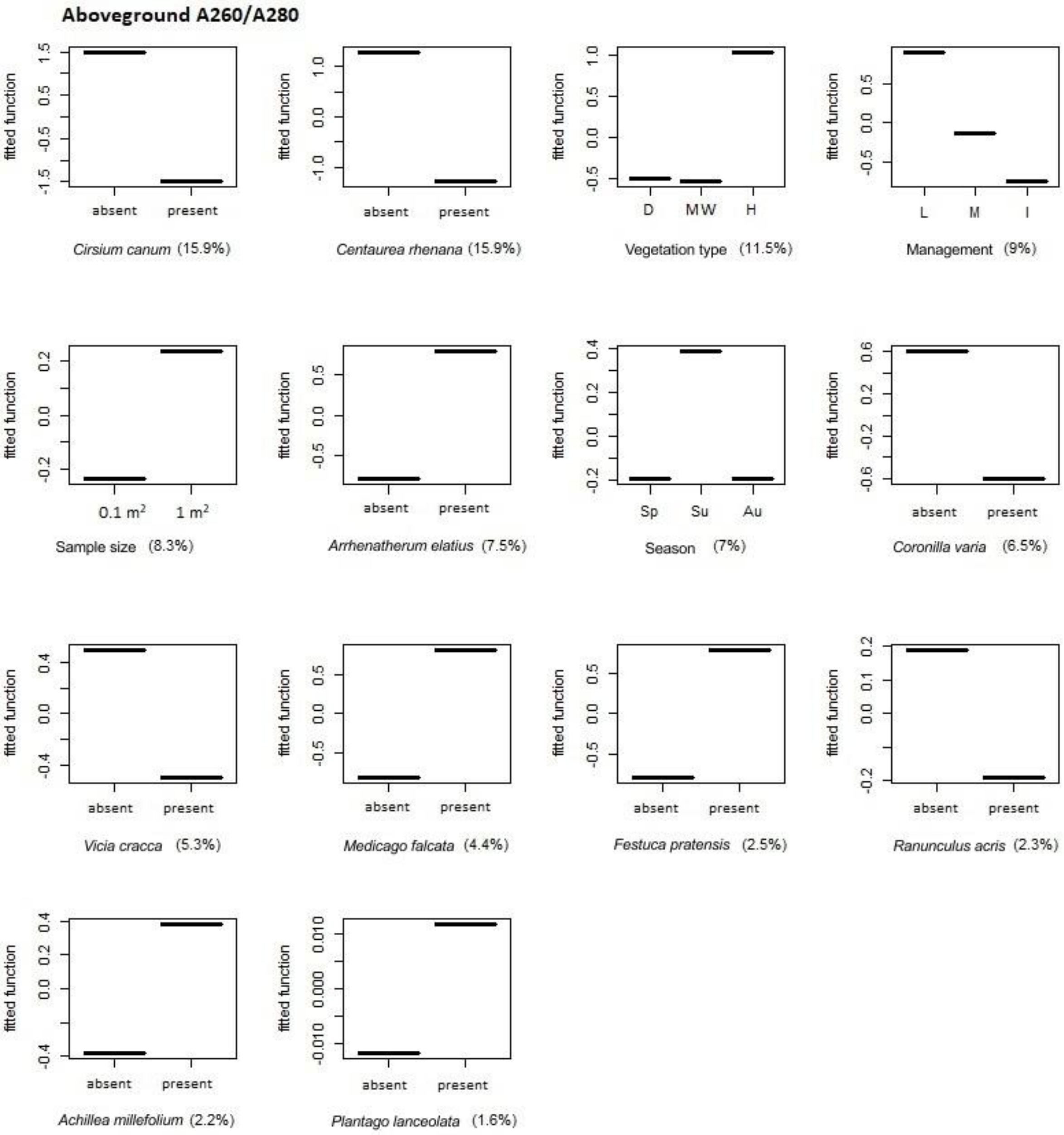

Fig. S5. Partial dependence plots showing the marginal relationships of aboveground DNA quality shown as ratio A260/A280 for all predictors of simplified BRT model.

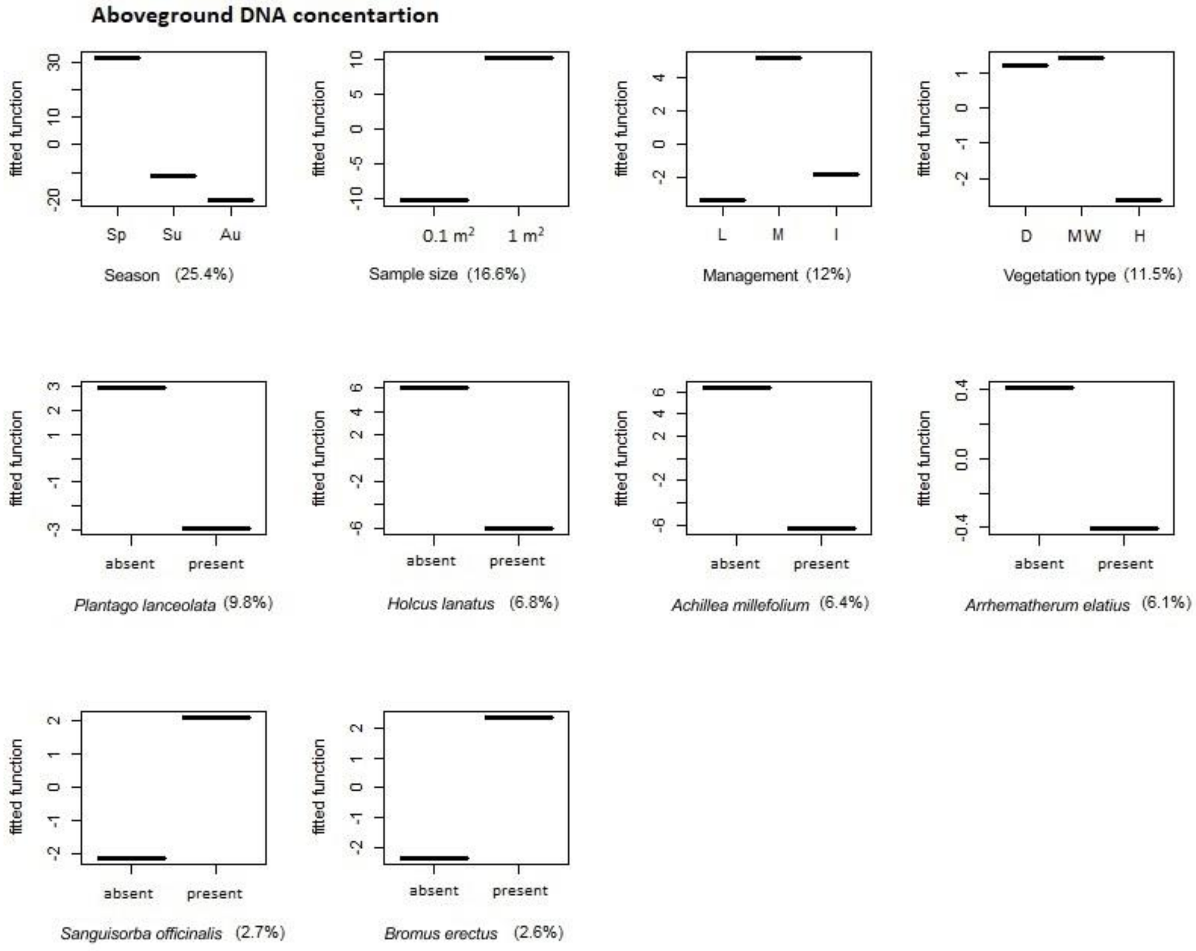

Fig. S6. Partial dependence plots showing the marginal relationships of aboveground DNA concentration for all predictors of simplified BRT model.
